# BioBBC: a multi-feature model that enhances the detection of biomedical entities

**DOI:** 10.1038/s41598-024-58334-x

**Published:** 2024-04-02

**Authors:** Hind Alamro, Takashi Gojobori, Magbubah Essack, Xin Gao

**Affiliations:** 1 Computer Science Program, Computer, Electrical and Mathematical Sciences and Engineering Division, King Abdullah University of Science and Technology (KAUST), Thuwal, Saudi Arabia; 2 Computational Bioscience Research Center (CBRC), King Abdullah University of Science and Technology (KAUST), Thuwal, Saudi Arabia; 3 College of Computing, Umm Al-Qura University, Mecca, Saudi Arabia

**Keywords:** Biomedical named entity recognition, Machine learning, Natural language processing, NER, BiLSTM, BioBERT, PubMedBERT, Computer science, Biological techniques, Bioinformatics

## Abstract

The rapid increase in biomedical publications necessitates efficient systems to automatically handle Biomedical Named Entity Recognition (BioNER) tasks in unstructured text. However, accurately detecting biomedical entities is quite challenging due to the complexity of their names and the frequent use of abbreviations. In this paper, we propose BioBBC, a deep learning (DL) model that utilizes multi-feature embeddings and is constructed based on the BERT-BiLSTM-CRF to address the BioNER task. BioBBC consists of three main layers; an embedding layer, a Long Short-Term Memory (Bi-LSTM) layer, and a Conditional Random Fields (CRF) layer. BioBBC takes sentences from the biomedical domain as input and identifies the biomedical entities mentioned within the text. The embedding layer generates enriched contextual representation vectors of the input by learning the text through four types of embeddings: part-of-speech tags (POS tags) embedding, char-level embedding, BERT embedding, and data-specific embedding. The BiLSTM layer produces additional syntactic and semantic feature representations. Finally, the CRF layer identifies the best possible tag sequence for the input sentence. Our model is well-constructed and well-optimized for detecting different types of biomedical entities. Based on experimental results, our model outperformed state-of-the-art (SOTA) models with significant improvements based on six benchmark BioNER datasets.

## Introduction

The number of biomedical publications is increasing rapidly. Currently, PubMed has more than 35 million abstracts for biomedical literature, with an average of one million new records added each year. Additionally, PubMed Central offers access to 9 million full-text articles^1^. This means that researchers need to sift through an impossibly large amount of literature/published articles to obtain valuable information. Moreover, new biomedical discoveries, experiments, and results are published in an unstructured form, making extracting relevant information time-consuming^2^. Consequently, researchers are now using biomedical text mining techniques to enhance this process^3^.

Named Entity Recognition (NER) is a form of information retrieval used in natural language processing (NLP). It is the task of automatically recognizing and locating entity mentions in a text and classifying them into predefined categories, such as person names, organizations, locations, etc. The NER task was first introduced in 1996 during the sixth Message Understanding Conference^4^ to identify specific terms and symbols. NER has since been used for several diverse NLP tasks, including relation extraction, knowledge graph construction, question answering, and machine translation^5^. NER can be approached as a sequence labeling problem wherein the objective is to assign a label to each term in a sentence based on predefined categories. There are different tagging choices for NER systems. Several annotation schemes have been used in the literature, including IO which annotates (Inside/Outside) entities, BIO (Beginning/Inside/Outside), and BIOES (Beginning/Inside/Outside/End/Single). The choice between tagging schemes often depends on the specific requirements of the NER task and the preferences of the researchers or practitioners.

Identifying biomedical domain-specific entities, such as genes, diseases, drugs, and so on, referred to as biomedical named entity recognition (BioNER), is particularly challenging due to several reasons, such as naming complexity (a mix of symbols and numbers in entity names), frequent occurrences of abbreviations, the problem of new entity names, and data privacy concerns^6–8^. Correctly identifying these entities is crucial for enhancing the quality of biomedical NLP applications, such as extracting drug-drug interactions^9^ and disease-gene relationships^10^. In the biomedical domain, the choice of the NER model depends on the specific research objectives, data characteristics, and computational constraints. Several model architectures have been particularly influential in the BioNER task, with early systems primarily based on dictionaries, rules, and machine learning (ML)^11–14^ and more recent systems using neural networks and deep learning (DL)^15,16^. DL methods can learn and extract useful features by creating embedding vectors. Recurrent Neural Networks (RNNs), particularly Bi-directional Long Short-Term Memory (Bi-LSTM) networks, are utilized as an encoder to extract sequence information and capture dependencies within the text. Conditional random fields (CRF)^17^ usually follow BiLSTM to assign the named entity labels. CRF is a method that can consider the correlation between neighboring labels. It can obtain the global optimal label chain for a given sequence. These capabilities of combining BiLSTM-CRF make it one of the preferred architectures used in NER systems.

Moreover, pre-trained language models have gained popularity in recent years due to their remarkable success and outstanding performance. For instance, Bidirectional Encoder Representations from Transformers (BERT)^18^ has made impressive progress in various natural language processing (NLP) tasks. In the field of biomedicine, numerous pre-trained models have been proposed. For instance, BioBERT^19^, BlueBERT^20^, and ClinicalBERT^21^ further extend the general domain language models with biomedical text. Moreover, the pre-trained models SciBERT^22^ and PubMedBERT^23^ construct a domain-specific vocabulary from scratch. SciBERT was pre-trained on scientific literature in computer science and biomedicine, while PubMedBERT was trained from scratch on biomedical literature.

In this paper, our focus is on improving the performance of BioNER by enriching the BERT-BiLSTM-CRF with multiple feature embedding. At first, to encode the input text, we generate multiple types of feature embeddings, including POS tag embeddings, char-level embeddings, and contextual word-level embeddings. For char-level embedding, we utilized a bidirectional LSTM (BiLSTM), while for word-level embedding, we employed the BERT and the data-specific embedding models. The outputs from the different embedding models are then concatenated and fed into a BiLSTM layer. The BiLSTM learns the relevant contextual information necessary for predicting named entities. Finally, the CRF will assign and output the best sequence of labels.

Although several studies have followed the BERT-BiLSTM-CRF architecture, they primarily rely on the automatically generated features of BERT. BioBBC, however, leverages additional knowledge by fusing different types of embeddings. Incorporating these varied embeddings has enhanced the model's capacity to capture relevant information for entity recognition, though it incurs significant computational costs. Consequently, it is crucial to carefully determine which combinations to use, ensuring they are strategically selected to enhance the model's performance effectively.

BioBBC exploits the robustness of this architecture and further improves it by utilizing the following approaches:

- Incorporating additional features to complement BERT embeddings, including three extra embeddings types: (1) syntactic features, (2) character embeddings, and (3) domain-specific word embeddings.
- Evaluating the impact of the concatenated input features by assessing the effectiveness of each component.
- Optimizing the architecture through learning and selecting different configurations to improve the model's expressiveness.

Our model follows the single learning approach, where we develop a separate model for each entity type. We present the performance results of our model based on eight benchmark BioNER datasets: NCBI-Disease^24^, BC5CDR-Disease^25^, BC5CDR-Chem^25^, BC4CHEMD^26^, BC2GM^27^, JNLPBA^28^, LINNAEUS^29^, and Species-800^30^.

## Related work

The earlier studies on BioNER systems were rule-based or dictionary-based approaches^11,12,31,32^. These systems have a simple structure but require up-to-date dictionaries and manually crafted feature sets. The main problem with traditional methods, commonly called the out-of-vocabulary problem (OOV), is that they cannot handle new words not seen during training. Thus, ML-based models were also applied to solve the task of NER. For instance, TaggerOne^33^ used a semi-Markov classifier for biomedical entity identification and linking. Other studies solved NER using techniques such as Support Vector Machine (SVM)^34,35^, Hidden Markov Models (HMM)^36,37^, and Structural Support Vector Machines (SSVM)^38^. However, a challenge in these methods arises with the requirement for manual feature extraction from raw data. Thus, neural networks and DL methods were recently also applied to BioNER^15,16^.

The primary advantage of DL methods is their capability to automatically extract useful features through embedding vectors, eliminating the need for manual feature extraction. Examples include the model introduced by Lample et al. that combines the word vector representation models, LSTMs and CRF, into a single method called BiLSTM-CRF^39^. Hong et al.^40^ proposed a DL label-label transition model named DTranNER. Crichton et al.^41^ also used the word token with its surrounding context words as the input for a model based on Convolutional Neural Networks (CNNs). Luo et al*.*^8^ proposed a document-level attention-based model for chemical NER. Moreover, the study^42^ improves the accuracy of entity recognition by combining LSTM, CRF, word embeddings, and char-level representation. Yoon et al^43^ developed the CollaboNet model that comprises multiple BiLSTM networks. Each network acts as a single task to recognize a specific entity type, resulting in more precise predictions. Later, Tong et al.^6^ developed MT-BioNER, which uses a multi-task learning approach to solve BioNER.

Recently, pre-trained language models have been applied in BioNER. Domain-specific BERT such as BioBERT^19^, SciBERT^22^, and PubmedBERT^23^ have significantly outperformed previous BioNER systems. Furthermore, several studies^44–46^ combined biomedical BERT with various ML and DL strategies and achieved state-of-the-art (SOTA) performances. For instance, BioByGANS^45^ used BioBERT with graph neural networks and solved BioNER as a node classification problem. Wang and Gu^47^ developed a Biaffine Layer on top of BERT-BILSTM, serving as a bidirectional mapping network for improved entity extraction and semantic information capture. Guan and Zhou^48^ proposed an enhanced BERT and improved sequence labeling performance through a word-pair classification strategy. Moreover, some studies, such as BioBERT-MRC^44^ and KaNER^49^ adopted the Machine Reading Comprehension (MRC) approach to solve BioNER.

In addition, syntactic features such as part-of-speech (POS tags), syntactic constituents, and dependency parsing have shown advantages in NLP downstream tasks. Syntactic features can help in improving the performance of BioNER. Specifically, the text of the biomedical domain is usually formal, consists of long sentences, and contains domain-specific terms. Thus, syntactic information can provide helpful information by analyzing the grammatical structure of sentences, which helps understand the relationship between the words and recognizing entities. In BioNER, several studies have used syntactic information to improve performance^8,45,50,51^. We improved this area by combining contextual and syntactic features using multi-feature embeddings. Our model learns the input using different representation types, including char-level, word-level, and POS tags features.

## Method

### Problem definition

Given an input sentence X = { x_1_,  x_2_, …, x_n_}, which is a sequence of words where xi represents the i-th word of the sentence and n represents the length of the sentence. Our NER model aims to predict a sequence of corresponding labels Y = {y_1_, y_2_, …, y_n_}, where yi represents the label of the word xi. The labels refer to a predetermined list of biomedical entity types.

### Model details

**Figure 1:**
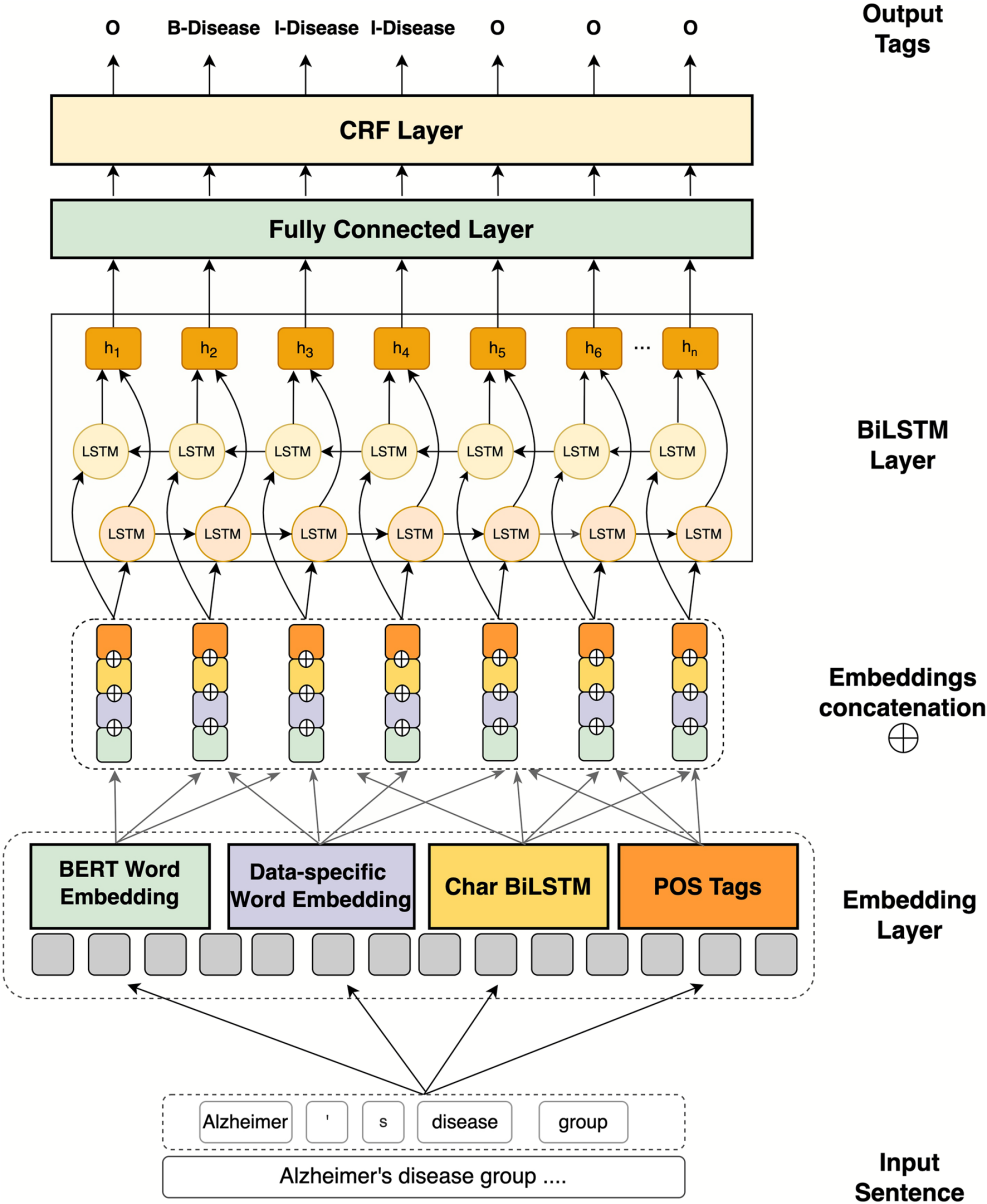
The architecture of the proposed model.

The proposed model is shown in Fig. 1. The model comprises three primary components: an Embedding layer, a BiLSTM layer, and a CRF layer. The embedding layer comprises four representation models: one for POS tags, one for char-level, and two for word-level. The POS tag embedding is obtained through one-hot encoding. The char-level representation is obtained through a BiLSTM layer, while the word contextual representations are obtained through a BERT layer and a data-specific embedding layer. These types of embeddings are then concatenated and fed to the BiLSTM layer to obtain additional syntactic and semantic feature representations. The output of the BiLSTM is fed into a fully connected layer, which passes the vectors to the CRF layer. The CRF layer identifies the best possible tag sequence for the input sentence.

#### Embedding layer

We use four representation methods to capture more information about the input text, i.e., POS tag embeddings, char-level embeddings, the data-specific embeddings, and the BERT embeddings. The four types of embeddings are concatenated to perform one long vector used as input to the successive layers, BiLSTM.

##### POS tags embedding

POS tags indicate the grammatical properties of words within a sentence. Examples of these parts of speech include nouns, pronouns, adjectives, determiners, verbs, adverbs, prepositions, conjunctions, and interjections. We used the NLTK Python library^52^ to extract the POS tags of the sentences. We employed a one-hot encoder to generate embeddings for the POS tags. The one-hot encoder is transformed into a lower dimension using *nn.Embedding(one-hot-size, emb_dim),* where *one-hot-size* represents the length of the one-hot vector, and the embedding size is set to 50.

##### Char-level embedding

We used the char-level representation to extract char-level features for each word in the text. Specifically, we passed each character in the input word through a BiLSTM layer, which converts the character into a vector representation. These vectors are combined for each word, generating a char-level representation of a word with a vector size of 50.

##### Word-level data-specific embedding

We utilized Flair^53^, an open-source Python library, to generate word-level, data-specific embeddings. Flair offers several NLP solutions, including a Flair language model trainer that can be employed to create custom embedding. This language model trainer produces word-level embeddings that are represented at the character level, meaning it represents words as sequences of characters contextualized by the surrounding text. This feature is valuable for addressing OOV words common in biomedical texts.

Accordingly, we first aggregated all the datasets we used in this study to generate our data-specific embeddings and then trained a Flair language model on this combined dataset. This process results in our data-specific embeddings. The Flair language model uses an LSTM layer to generate the embeddings, and we created both forward and backward embeddings of the data. Thus, the output from this data-specific embedding consists of two vectors, one forward and one backward; these vectors will be concatenated with other embedding vectors in the embeddings concatenation stage.

##### Word-level BERT embedding

We obtained the second word-level representation using BERT, a pre-trained language model that uses multilayer bidirectional transformer encoders to generate language representations. The architecture of BERT uses 12 layers of transformers block with a hidden size of 768 and 12 self-attention heads and was trained on English text sourced from BookCorpus and Wikipedia. In this project, we used a domain-specific variation of the BERT model called PubMedBERT^23^. PubMedBERT is a pre-trained language model based on the architecture of BERT, trained from scratch on PubMed abstracts and full-text articles from PubMed Central. We used the version trained only on the abstracts, ‘Microsoft/BiomedNLP-PubMedBERT-base-uncased-abstract’ released by HuggingFace.

To prepare sentences for encoding by the BERT model, we must follow the input format of the BERT. First, we add the special tokens, [CLS] and [SEP], to each sentence’s beginning and end, respectively. Also, if the input sentence is shorter than the chosen max_length of 128, it will be padded to ensure that all input sentences are of equal length. The output of BERT is a hidden state vector of size 768 for each token in the input sequence.

##### Embeddings concatenation

The output vectors obtained from the different embeddings (POS tags, char-level, data-specific, and BERT) are concatenated to perform one long vector. This vector is used as the input into the next layer.

#### BiLSTM layer

Long Short-Term Memory (LSTM)^54^ is a type of recurrent neural network (RNN) that learns long-term dependencies between patterns in sequence data, making it widely used in sequence labeling problems. The structure of LSTM includes a memory module that helps to keep track of the seen information from the sequence data that has been processed. This structure, called forward LSTM, processes input in one direction (e.g., left to right).

The Bidirectional LSTM (BiLSTM)^55^ combines forward LSTM and a backward LSTM. Thus, it can capture information from both preceding and succeeding words in a sequence, which allows a more comprehensive understanding of context and semantic information and facilitates learning of the dependencies between contexts. In BiLSTM, the representation of each word in the input sequence is calculated twice: once from left to right (ht →) and once from right to left (ht ←). These two representations are then concatenated, ht = [ht → ; ht ←], to produce the final vector representation of each word.

In our model, the input to the BiLSTM is the output of the Embedding layer, which consists of a sequence of vectors. The BiLSTM takes these vectors and calculates the forward representation (h1 → , h2 → , …, hn →) and backward representation (h1 ← , h2 ← , …, hn ←) using the forward LSTM and backward LSTM, respectively. The dimension size of each LSTM layer is 256. Then, these representations are concatenated for each word (e.g., h1 = [h1 → ;h1 ←]), producing an output vector with a size of 512 (2*256). The final output of BiLSTM is the complete representation of the sentence (h1, h2, …, hn).

The output of the BiLSTM is mapped from its original dimension of 512 to a k-dimension using a fully connected layer. In this case, k represents the number of labels present in the dataset.

#### CRF layer

The output vector from the BiLSTM layer can be used directly with a Softmax layer to make independent tagging decisions for each output. This approach may produce an invalid sequence of labels, such as "O, I-Disease, I-Disease, …” where in BIO format, the tag "I" must follow a "B" tag^56^. Thus, it is necessary to learn dependencies across output labels in sequence labeling tasks.

CRF solves this issue by learning the relationships between adjacent tags in a sentence, ensuring that the predicted tag sequences are valid. During training, the CRF layer learns constraints and transitional probabilities to identify the best possible tag sequence for the given sentence. Some constraints in BIO tagging format include that the label of the first word in a sentence should start with the tag of "B" or "O", but not "I".

**Figure 2:**
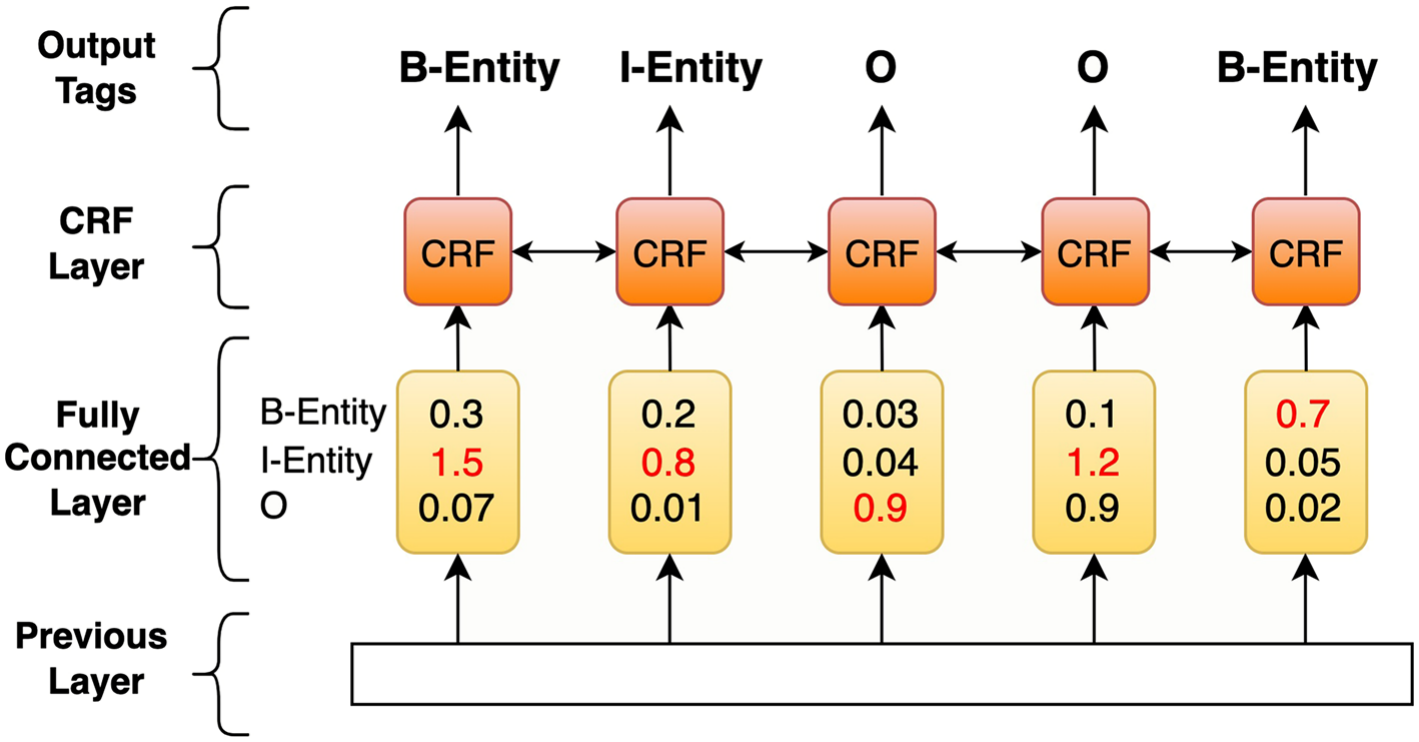
The emission scores from previous layer are the input to CRF.

CRF uses two types of scores: emission scores (P) and transition scores (T). The emission scores are the previous layer's output, representing the predicted scores for each label (see Fig. 2). On the other hand, transition scores are learned during the training process. A transition score represents the probability of transitioning from the tag of word x_i_ to the tag of x_i + 1_ in a given sequence X = {x_1_,  x_2_, …, x_n_}.

Mathematically, given a sentence of text X = { x_1_, x_2_, …, x_n_} and an output label sequence Y = {y_1_, y_2_, …, y_n_}, a CRF calculates the score of labels for the sequence using the following equation:

$$Score\left( {X,Y} \right) = \mathop \sum \limits_{i = 1}^{n} P_{{i,y_{i} }} + \mathop \sum \limits_{i = o}^{n} T_{{y_{i} ,y_{i + 1} }}$$

where P is a matrix of scores with dimensions n × k, where n represents the length of the sentence and k represents the number of distinct tags. $P_{i,j}$ represents the score assigned to the jth tag for the ith word. T is a transition matrix where $T_{{y_{i} ,y_{i + 1} }}$ represents the probability of transitioning from label i to label i + 1.

The final decoding sequence is determined by selecting the highest predicted score obtained through the Viterbi algorithm.

$$Y^{*} = arg max\left( {Score\left( {X,Y} \right)} \right)$$

## Experiments

### Datasets

- *NCBI-Disease*^24^ is a dataset fully annotated for diseases at both the mention and concept levels. The dataset includes 793 PubMed abstracts, 6,892 mentions of diseases, and 790 distinct disease concepts.
- *BC5CDR*^25^ is a dataset created for the BioCreative V challenge. The dataset contains two sub-datasets: BC5CDR-Disease and BC5CDR-Chemical. We used these sub-datasets to evaluate diseases and chemicals, respectively.
- *BC4CHEMD*^26^ is a dataset used for the BioCreative IV Chemical Compound and Drug Name Recognition task. The dataset comprises 10,000 abstracts of PubMed publications annotated for Chemical/Drug entities.
- *BC2GM*^27^ is a dataset created for the BioCreative II Gene Mention Recognition task. This dataset consists of 20,000 sentences from PubMed annotated with over 24,000 gene mentions.
- *JNLPBA*^28^ is a biomedical corpus developed for a joint workshop on NLP in biomedicine and its applications. This dataset comprises 2,000 PubMed abstracts aimed at identifying entities related to molecular biology, such as proteins, DNA, RNA, cell lines, and cell types. Following^19^ and previous studies, we did not use cell-type and cell-line entity tags from JNLPBA. Instead, we focused solely on identifying protein, DNA, and RNA entities, which we annotated as Gene.
- *LINNAEUS*^29^ is a biomedical corpus for species entity recognition and normalization. It consists of 4259 species entities annotated manually from 100 PMC full-text documents.
- *Species-800*^30^ is a manually annotated corpus for species entities, annotated from 800 PubMed abstracts.

For all datasets, we used the preprocessed BIO versions provided by^19^. The specifics of each dataset are outlined in Table 1.

**Table 1:** Number of sentences in Training, Validation, and Testing files in each dataset.

Dataset	Entity type	Number of sentences			
		Training	Validation	Testing	Total
NCBI-disease	Disease	5701	634	940	7275
BC5CDR-Disease	Disease	8226	915	4797	13,938
BC5CDR-Chem	Chemical/Drug	8226	915	4797	13,938
BC4CHEMD	Chemical/Drug	55,188	6133	26,364	87,685
BC2GM	Gene/Protein	13,583	1510	5038	20,131
JNLPBA	Gene/Protein	16,691	1855	3856	22,402
LINNAEUS	Species	14,411	1602	7142	23,155
Species-800	Species	5906	657	1630	8193

### Tagging schema

In our study, for all datasets, we used the pre-processed versions provided by^19^. The provided dataset was in the BIO form. The BIO tagging scheme allows for the representation of multi-token entities and enables the model to distinguish between the beginning and continuation of entities within a sequence. This facilitates the training and evaluation of NER models by providing clear boundaries for each entity type. Thus, we subsequently used this schema to make a fair comparison with the previous models.

**Figure 3:**
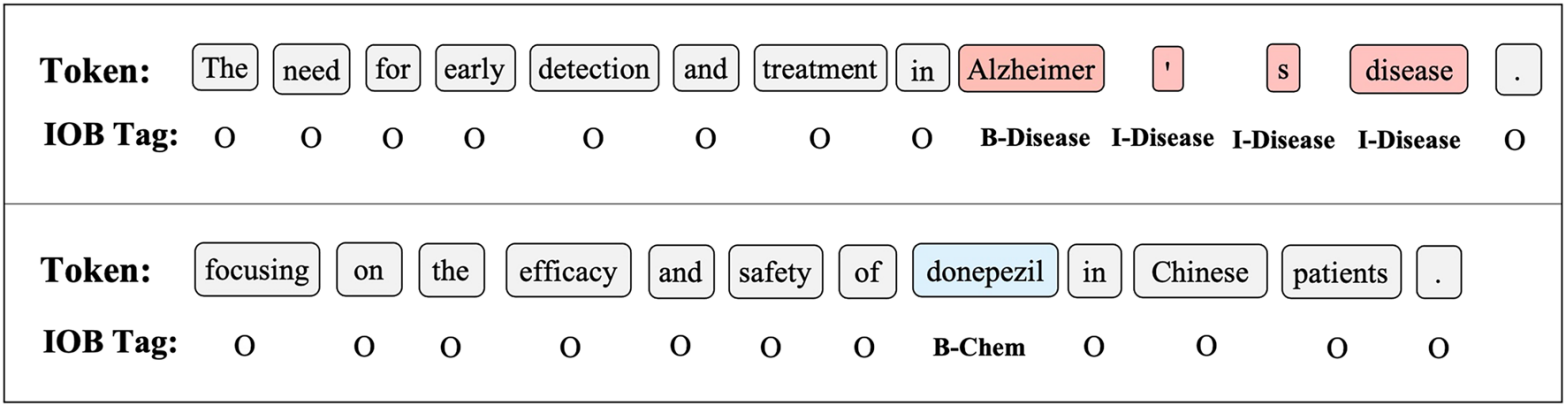
Example of BIO tagging format.

In BIO labeling format, the term "B-Entity" (beginning) indicates that it is the first word of an entity, while "I-Entity" indicates that it is a middle or last word of an entity. In contrast, the label "O" (which stands for "Outside" or "Other") indicates that the word does not belong to any named entity. The term "entity" refers to any biomedical entity, including disease, chemical/drug, gene, and species targeted in this study. Figure 3 provides examples of the BIO tagging.

### Experimental settings

Following previous works^6,19,43–45^, we train the final models by merging the training and development sets and using a 10% split of this merged set for validation, while the provided testing file was used for evaluation. Table 1 provides the number of sentences in each set.

Several factors, such as dataset characteristics and available memory and resources, influence the hyperparameter selection process. In BioBBC, we established a range of parameter spaces, as listed in Table 2. We tuned these parameters on the validation set to select the optimal choices. For the maximum sentence length, we chose 128 over 256 for two primary reasons: firstly, the performance difference between the two lengths was negligible across all models, and secondly, 128 is more memory-efficient. Consequently, we excluded some long sentences (approximately 20) from BC4CHEMD. The learning rate selection had the most substantial impact, with optimal values varying across datasets. The batch size was set to 64, except for Linnaeus and BC4CHEMD, where it was adjusted to 32. The hidden state size in the BiLSTM was set to 256, as it offered an improvement over 128, while 512 caused memory issues. The maximum number of epochs was set to 100. The selected configuration for each model is shown in Table 2.

**Table 2:** Hyperparameters used for each model.

Hyperparameter	Max-length	Batch-size	Learning-rate	BiLSTM hidden layer size
Search Space	(128, 256)	(16, 32, 64, 128)	(0.1, 1e−2, 1e−3, 3e−3, 3e−5, 5e−2, 5e−3, 5e−4, 5e−5)	(128, 256, 512)
Dataset				
NCBI-disease	128	64	5e−5	256
BC5CDR-Disease	128	64	5e−3	256
BC5CDR-Chem	128	64	5e−3	256
BC4CHEMD	128	32	1e−3	256
BC2GM	128	64	3e−5	256
JNLPBA	128	64	3e−5	256
LINNAEUS	128	32	5e−5	256
Species-800	128	64	5e−5	256

The models were implemented using PyTorch version 1.13.1 and Transformers version 4.27.4.

### Evaluation metrics

For the evaluation, we utilized precision (P), recall (R), and F1-score (F1). Precision measures the model's ability to identify positive entities accurately. It is the ratio of correctly classified positive samples (True Positive) to the total number of classified positive samples. The higher the precision, the more accurate the prediction. Recall measures the model's ability to identify all positive instances correctly. This refers to the ratio of correctly predicted positive samples to the total number of positive samples. The higher the recall, the more positive samples are detected. The F1-score represents the harmonic mean of precision and recall. Precision, recall, and F1-score are calculated using the following formulas:

$$P = \frac{TP}{{TP + FP}}$$

$$R = \frac{TP}{{TP + FN}}$$

$$F1 = \frac{2 * P * R}{{P * R}}$$

In this context, TP refers to True Positive, which indicates the number of positive classes correctly predicted as positive. FP stands for False Positive, indicating the number of negative classes incorrectly predicted as positive. On the other hand, FN stands for False Negative, indicating the number of positive classes incorrectly predicted as negative. We considered strict matching at the entity level, where the predicted entity's type and boundary must be correct. Thus, a true positive is only counted for an entity with multiple tokens if the entire entity, including all its tokens, is captured. Here, we used Seqeval, a Python library for evaluating sequence labeling [https://github.com/chakki-works/seqeval].

## Results and discussion

### Comparison with existing models

To evaluate the performance of our model, we compared the model with different baseline models, including TaggerOne^33^, BiLSTM-CRF^39^, CollaboNet^43^, BioBERT^19^, DTranNER^40^, BioBERT-MRC^44^, MT-BioNER^6^, BioByGANS^45^, KaNER^49^, and PAMDFGA^48^. Furthermore, we used diverse benchmark datasets, including disease datasets (NCBI-Disease and BC5CDR-Disease), Chemical/Drug datasets (BC5CDR-Chem and BC4CHEMD), and genes datasets (BC2GM and JNLPBA), and species datasets (LINNAEUS and Species-800) to demonstrate performance more generically.

Tables 3, 4, 5 and 6 summarize the performance results of the existing BioNER models for comparison. We used **bold** and underline to indicate the best and second-best performance scores, respectively. Overall, our proposed system outperformed the baseline model in almost all the datasets. This might result from how we designed our model so that the embedding layer learns enriched features of the input text, resulting in a better understanding of the text from different aspects, including POS tag, char-level, and word-level. Specifically, Table 3 shows our model achieved the best scores overall, except for the recall score in NCBI-Disease, where BioByGANS achieved a better result. On BC5CDR-Disease, though, our model improved the F1-score by 1.32% compared to the previous best score. Also, for the Chemical/Drug entities, shown in Table 4, our model achieved the best score of 0.9422 (with an improvement of 1.25%) on the BC4CHEMD dataset and the second-best F1 on the BC5CDR-Chem. Our model also achieved significant improvement using the Gene/protein datasets (see Table 5), with best scores of 0.8912 (which represents an improvement of 3.64%) in BC2GM and 0.7939 in JNLPBA.

**Table 3:** Performance comparison for the disease entity. The best scores are Bold and the second best are underlined.

Method\Dataset	NCBI-disease			BC5CDR-disease		
	P	R	F1	P	R	F1
TaggerOne^33^	0.8510	0.8080	0.8290	0.8520	0.8020	0.8260
BiLSTM-CRF^39^	0.8611	0.8549	0.8580	0.8760	0.8625	0.8692
CollaboNet^43^	0.8548	0.8727	0.8636	0.8561	0.8261	0.8408
DTranNER^40^	0.8821	0.8904	0.8862	0.8675	0.8770	0.8722
BioBERT-MRC^44^	0.8967	0.9042	0.9004	0.8861	0.8707	0.8783
MT-BioNER^6^	0.8890	0.9094	0.8991	–	–	–
BioByGANS^45^	0.8999	**0.9320**	0.9157	0.8669	0.8882	0.8774
BioBERT^19^	0.8822	0.9125	0.8971	0.8647	0.8784	0.8715
PAMDFGA^48^	0.8976	0.9135	0.9055	0.8711	0.8795	0.8753
KaNER^49^	0.9043	0.9207	0.9124	–	–	–
**Ours**	**0.9057**	0.9278	**0.9166**	**0.8870**	**0.8961**	**0.8915**

**Table 4:** Performance comparison for Chemical/Drug entity.

Method\Dataset	BC5CDR-Chem			BC4CHEMD		
	P	R	F1	P	R	F1
TaggerOne^33^	0.9420	0.8880	0.9140	–	–	–
BiLSTM-CRF^39^	0.9282	0.8852	0.9062	0.9131	0.8773	0.8948
CollaboNet^43^	0.9426	0.9238	0.9331	0.9078	0.8701	0.8885
DTranNER^40^	0.9428	0.9404	0.9416	0.9194	0.9204	0.9199
BioBERT-MRC^44^	0.9437	0.9400	0.9419	0.9389	0.9196	0.9292
MT-BioNER^6^	0.9329	0.9469	0.9398	–	–	–
BioByGANS^45^	0.**9453**	0.9495	**0.9474**	0.9342	0.9252	0.9297
BioBERT^19^	0.9368	0.9326	0.9347	0.9280	0.9192	0.9236
PAMDFGA^48^	0.9366	0.9467	0.9416	0.9174	0.9337	0.9255
**Ours**	0.9404	**0.9534**	0.9469	**0.9399**	**0.9445**	**0.9422**

**Table 5:** Performance comparison for Gene entity.

Method\Dataset	BC2GM			JNLPBA		
	P	R	F1	P	R	F1
TaggerOne^33^	–	–	–	–	–	–
BiLSTM-CRF^39^	0.8157	0.7948	0.8051	0.7135	0.7574	0.7348
CollaboNet^43^	0.8049	0.7899	0.7973	0.7443	0.8322	0.7858
DTranNER^40^	0.8421	0.8484	0.8456	–	–	–
BioBERT-MRC^44^	0.8704	0.8398	0.8548	0.7596	0.8213	0.7893
MT-BioNER^6^	0.8442	0.8514	0.8478	–	–	–
BioByGANS^45^	0.8497	0.8532	0.8515	0.7269	0.8454	0.7816
BioBERT^19^	0.8432	0.8512	0.8472	0.7224	0.8356	0.7749
PAMDFGA^48^	0.8543	0.8547	0.8545	–	–	–
KaNER^49^	–	–	–	**0.7832**	0.7937	0.7884
**Ours**	**0.8944**	**0.8881**	**0.8912**	0.7347	**0.8635**	**0.7939**

**Table 6:** Performance comparison for Species entity.

Method\Dataset	LINNAEUS			Species-800		
	P	R	F1	P	R	F1
BioByGANS^45^	**0.9391**	**0.8825**	**0.9099**	0.7153	0.7883	0.7501
BioBERT^19^	0.9077	0.8583	0.8824	**0.7280**	0.7536	0.7406
**Ours**	0.9064	0.8612	0.8732	0.7121	**0.8031**	**0.7549**

Tables 3, 4, 5 and 6 show BioByGANS is the most competitive model to ours, as it achieved higher recall in NCBI-Disease and higher precision and F1-score in BC5CDR-Chem than our model. However, for no dataset does BioByGANS outperform our model in all metrics, except LINNAEUS (see Table 6), whereas our model outperformed BioByGANS in all metrics using the BC5CDR-Disease, BC4CHEMD, BC2GM, JNLPBA, and Species-800 datasets. Although BioByGANS uses a different approach than our model, we see that the shared property between BioByGANS and our model is that both models capture the POS tags features of the input sentence, which suggests that capturing the contextual and syntactic features improves the performance of the BioNER.

### Effect of using different pre-trained models

To investigate the impact of different BERT models, we compared four pre-trained language models from the biomedical domain. All four models, BioBERT, ClinicalBERT, SciBERT, and PubMedBERT, were downloaded from the HuggingFace website (https://huggingface.co/models).

*BioBERT*^19^ is based on the BERT model^18^, with further pre-training on biomedical scientific texts including PubMed abstracts (PubMed) and PubMed Central full-text articles (PMC). We used the “biobert-v1.1 (+ PubMed, Cased)” variant of BioBERT.

*ClinicalBERT*^21^ is also based on the BERT model, with further pre-training on biomedical domain-related clinical notes. We used the “emilyalsentzer/Bio_ClinicalBERT” variant of ClinicalBERT.

*SciBERT*^22^ is trained on 1.14 M full-text scientific papers from Semantic Scholar^57^ (18% papers from the computer science domain and 82% from the biomedical domain). SciBERT builds a domain-specific vocabulary (scivocab) from scratch to best match the training corpus. We used the “scibert-scivocab-uncased” variant of SciBERT.

*PubMedBERT*^23^ is trained from scratch and generates its own vocabulary. The pretraining corpus comprises 14 million PubMed abstracts with 3 billion words, and it also has another version that includes PMC full text articles, which increased the pretraining corpus to 16.8 billion words. PubMedBERT is the most recent pre-trained language model in the biomedical domain. We used the abstract-only variant of PubMedBERT “microsoft/BiomedNLP-PubMedBERT-base-uncased-abstract”.

**Figure 4:**
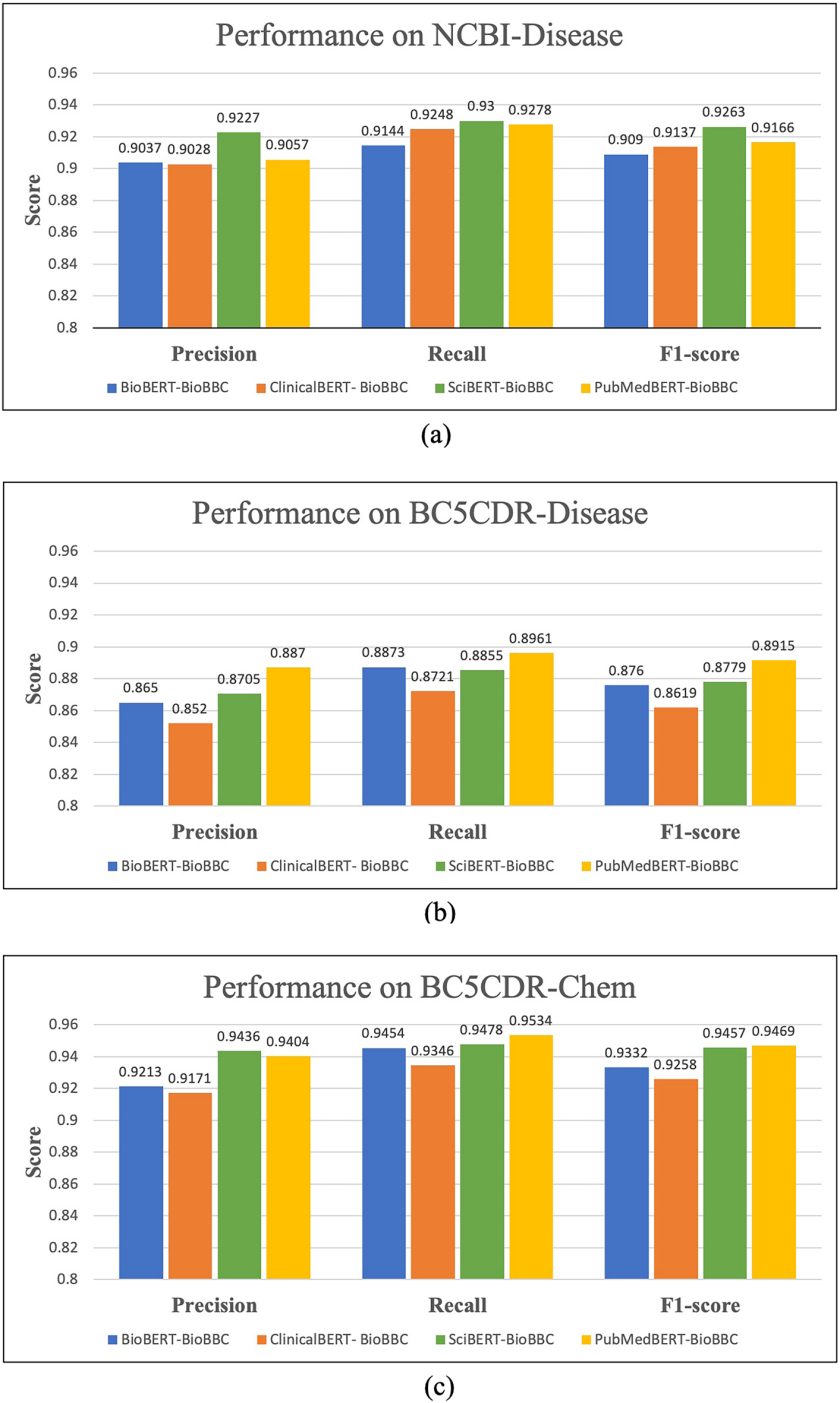
Performance comparison of using different BERT models with the (**a**) NCBI-Disease, (**b**) BC5CDR-Disease, and (**c**) BC5CDR-Chem datasets.

Figure 4 provides the models' performance results of each of the four pre-trained language models using NCBI-Disease, BC5CDR-Disease, and BC5CDR-Chem, respectively. The results show that PubMedBERT and SciBERT outperformed other models in this task. PubMedBERT achieved the highest scores for the BC5CDR-Disease and BC5CDR-Chem datasets, while for the NCBI-Disease dataset, SciBERT demonstrated superior performance. One reason could be that both ClinicalBERT and BioBERT use the same vocabulary as the general BERT, whereas SciBERT and PubMedBERT build domain-specific vocabularies from scratch. On the other hand, ClinicalBERT performed the worst for all the datasets used. This result corroborates Gu et al.^23^ findings and their suggestion that BERT models pre-trained on clinical notes are not well suited for BioNER tasks.

Although the differences in performance between PubMedBERT and SciBERT are small, we will mainly select PubMedBERT over SciBERT because it was trained on a larger biomedical domain, increasing its ability to cover more biomedical vocabulary. Specifically, the more extensive PubMedBERT vocabulary enhances the vocabulary coverage because it is more domain-specific, which further improves the performance. This is important for improving the performance because if the model does not recognize the term. In that case, it will be divided into small sub-words by the tokenizer, reducing its chance of being correctly identified in its biomedical class. In contrast, if the term is already included in the models' vocabulary, it will more likely be correctly recognized.

### Ablation study

We further conducted an ablation study to better understand each component's importance in our proposed model. We used the NCBI-Disease and BC5CDR-Disease datasets in this experiment.

Table 7 shows that the most impactful component is the PubMedBERT embeddings, while the data-specific embedding is the least impactful indicating the effectiveness of domain-specific BERT language models. The reason the data-specific embedding had the least impact may be because domain-specific BERT covers most of the information it provides. That is, the PubMedBERT model was trained on PubMed articles, which is the primary source of the experimental datasets.

**Table 7:** The ablation experiments.

Models	NCBI-disease			BC5CDR-disease		
	P	R	F1	P	R	F1
Embedding layer + BiLSTM + CRF	**0.9057**	**0.9278**	**0.9166**	**0.8870**	**0.8961**	**0.8915**
Without POS Tags	0.8856	0.8929	0.8892	0.8762	0.8945	0.8853
Without CharEmbedding	0.8962	0.8721	0.8840	0.8764	0.8930	0.8846
Without Data-specific Embedding	0.8991	0.9072	0.9031	0.8789	0.8974	0.8881
Without PubMedBERT Embedding	0.8636	0.8670	0.8653	0.8536	0.8570	0.8553
Without BiLSTM layer	0.9029	0.9183	0.9105	0.8802	0.8945	0.8873
Without CRF layer	0.8832	0.9174	0.9000	0.8800	0.8929	0.8864

Moreover, we found that removing the char-level embedding had a drop of 3.26% in F1-score in NCBI-Disease and a drop of 0.69% in BC5CDR-Disease. We also found that removing the POS tags embedding affects performance, resulting in a drop of 2.74% in NCBI-Disease and 0.62% in BC5CDR-Disease, indicating these components' importance in improving the model's performance.

Furthermore, removing the BiLSTM layer results in only a slight decrease in score. The reason could be that the BiLSTM is already used to produce the char-level and the data-specific embeddings; thus, most of the information that BiLSTM addresses is already gained in the embedding layer.

We also observed that the difference in the results is more noticeable in the NCBI-Disease dataset. One reason may be related to the size of the testing file. In NCBI-Disease, the test file includes only 940 sentences, whereas, in BC5CDR-Disease, the testing file contains more than 4700 sentences. Thus, even small changes in the results may impact the smaller testing size more.

Finally, in all cases of the ablation study, we observed that the model's performance was slightly degraded in each metric, indicating the critical impact of each component of the overall model.

Note, to further demonstrate the improvements that our model brings to BioNER, we conduct a case study comparing BioBBC to an existing online BioNER tool, PubTator3 [https://www.ncbi.nlm.nih.gov/research/pubtator3/]. We show examples of single sentences, large text with multiple sentences, and instances of error cases generated by BioBBC in the Supplementary Material.

## Limitations and concluding remarks

We developed a DL, end-to-end model named BioBBC to improve BioNER. Our model uses multi-feature embeddings to represent the input text, including char-level, word-level, and POS tags features. For the word-level, we used contextual features by PubMedBERT and data-specific features, which are embeddings generated for our datasets. We evaluated our approach using benchmark datasets for biomedical entities of diseases, chemicals, genes, and species types. The experimental results showed that BioBBC outperformed the existing BioNER model in terms of the F1-score on six out of eight benchmark datasets. Moreover, our case studies show the importance of syntactic and semantic learning in our model. Specifically, through several examples, our models show better performance in recognizing more biomedical entities and understanding the structure of the text, which results in more accurate entity detection.

While our model successfully recognizes biomedical entities, it does have some limitations. Firstly, it does not encompass all types of biomedical entities. For example, we did not include Phenotype in our study. However, most of the Phenotypes are covered under the disease entity type, for which we utilized two datasets including NCBI-Disease and BC5CDR. Secondly, the syntactic information extracted by the NLTK library may contain errors due to the specificity of biomedical text compared to general domain text, which could impact our model's performance. In addition, while enriching the model with extra knowledge through the BERT and data-specific embeddings, they may cause some ambiguity in the capturing of the entities.

In future work, we plan to expand the model to a multi-task learning approach that combines several datasets into one model. We will also include additional biomedical entity types, such as phenotypes, variants, and cell lines. Furthermore, we aim to explore more advanced syntactic and linguistic features specifically designed for the biomedical domain. Moreover, we intend to leverage Large Language Models (LLMs) like GPT-3 and its successors in BioNER to take advantage of the advancements in this field.

### Supplementary Information

Supplementary Information.

## Data Availability

The datasets used in this study are publicly available at https://github.com/dmis-lab/biobert. The trained models will be available at https://github.com/HindAlamro/BioBBC.
