# Supplementary material for: BioBBC: a multi-feature model that enhances the detection of biomedical entities
Source: Sci Rep. 2024 Apr 2;14:7697. doi: 10.1038/s41598-024-58334-x (PMC10987643; doi:10.1038/s41598-024-58334-x)
Supplement: Supplementary file 1 — Supplementary Information. [file 41598_2024_58334_MOESM1_ESM.pdf]

Supplementary Material

BioBBC: A Multi-feature Model that Enhances the Detection of Biomedical Entities

Case Study

To further demonstrate the improvements that our model brings to BioNER, we conduct a case study comparing BioBBC to an existing online BioNER tool, PubTator3 [https://www.ncbi.nlm.nih.gov/research/pubtator3/]. We show examples of single sentences, large text with multiple sentences, and instances of error cases generated by BioBBC

- ***Example of sentences***

Here, we compare our model BioBBC to the PubTator tool using examples of sentences. Table S1 displays the results. In case 1, the models should recognize entities of disease type. PubTator could not recognize the entity's boundary as it missed the token "congenital", whereas BioBBC correctly identified the multi-token entity "congenital DM". In the second case, the example contains three multi-token disease entities. PubTator failed to recognize the third entity, "prion diseases", while BioBBC correctly recognized all the entities with the correct boundary. In case 3, while the PubTator recognized the chemical entities "Allopurinol" and "Thioctic Acid", it failed to recognize their abbreviations "ALO" and "THA". At the same time, BioBBC successfully detects the chemical names' entities and their abbreviations' entities. Case 4 contains six chemical entities. While PubTator detects most of the entities, it failed to detect the multi-token chemical entity name "thiobarbituric-acid-reactive-substances" by ignoring the entity and all its parts. BioBBC, on the other hand, detected all the entities appearing in the sentence. In case 5, PubTator also could not recognize the entity's boundary as it did not pick up the token "prepro", whereas our system correctly recognizes this token. Here our model learned the relations between "prepro" and "AVP-NPII" by considering the syntactic and semantic features of the sentence. In case 6, PubTator failed

to recognize the gene's entity, "glucocorticoid receptors", whereas our model correctly recognized the entity and its abbreviation, "GR".

We further compared our model with PubTator3 [https://www.ncbi.nlm.nih.gov/research/pubtator3/] and found that, with the new version, it now accurately detected 3 out of the 6 listed examples. Specifically, PubTator3 correctly detects the tokens in the first three sentences but fails to do so in the remaining sentences where it still gives the same output as its previous version, underscoring areas where our model maintains superior performance over PubTator.

These examples show the robustness of BioBBC and its ability to better learn the syntactic and semantic information about the sentence, which extends to its ability to better recognize the abbreviation of the entities as well.

Table S1. Example of test sentences.

Case	Entity Type	Model	Sample
1.	Disease	PubTator	..transmission of congenital **DM** is rare and preferentially occurs with onset of **DM**..
		PubTator3	..transmission of **congenital DM** is rare and preferentially occurs with onset of **DM**..
		BioBBC	..transmission of **congenital DM** is rare and preferentially occurs with onset of **DM**..
2.	Disease	PubTator	such as **Alzheimer's disease**, **amyotrophic lateral sclerosis**, the prion diseases, and…
		PubTator3	such as **Alzheimer's disease**, **amyotrophic lateral sclerosis**, the **prion diseases**, and…
		BioBBC	such as **Alzheimer's disease**, **amyotrophic lateral sclerosis**, the **prion diseases**, and…
3.	Chemical/Drug	PubTator	the binary mixture of **Allopurinol** (ALO) and **Thioctic Acid** (THA) . objective : a comprehensive stability - indicating HPLC - DAD procedure has been executed for concurrent analysis of ALO and THA
		PubTator3	the binary mixture of **Allopurinol** (ALO) and **Thioctic Acid** (THA) . objective : a comprehensive stability - indicating HPLC - DAD procedure has been executed for

			concurrent analysis of **ALO** and **THA**
		BioBBC	the binary mixture of **Allopurinol** (**ALO**) and **Thioctic Acid** (**THA**) . objective : a comprehensive stability - indicating HPLC - DAD procedure has been executed for concurrent analysis of **ALO** and **THA**
4.	Chemical/Drug	PubTator	Then, the brain homogenates content of thiobarbituric-acid-reactive-substances (**TBARS**), **4-Hydroxy-2-nonenal** (**4-HNE**) and **acetylcholine** (**ACh**)/**acetylcholine**…
		PubTator3	Then, the brain homogenates content of thiobarbituric-acid-reactive-substances (**TBARS**), **4-Hydroxy-2-nonenal** (**4-HNE**) and **acetylcholine** (**ACh**)/**acetylcholine**…
		BioBBC	Then, the brain homogenates content of **thiobarbituric-acid-reactive-substances** (**TBARS**), **4-Hydroxy-2-nonenal** (**4-HNE**) and **acetylcholine** (**ACh**)/**acetylcholine**…
5.	Gene	PubTator	found in both the signal peptide of the prepro-**AVP-NPII** precursor and within **NPII** itself
		PubTator3	found in both the signal peptide of the prepro-**AVP-NPII** precursor and within **NPII** itself
		BioBBC	found in both the signal peptide of the **prepro-AVP-NPII** precursor and within **NPII** itself
6.	Gene	PubTator	The study demonstrated a decreased level of glucocorticoid receptors (GR) in peripheral blood lymphocytes
		PubTator3	The study demonstrated a decreased level of glucocorticoid receptors (GR) in peripheral blood lymphocytes
		BioBBC	The study demonstrated a decreased level of **glucocorticoid receptors** (**GR**) in peripheral blood lymphocytes

- ***Example of paragraph***

To further show the robustness of our system, we also conducted a case study using long paragraphs taken from two studies[1,2]. Since we have separate models for each entity type, we conducted this experiment by sequentially inputting the text into three models trained by NCBI-Disease, BC5CDR-Chem, and BC2GM. We then merged the resulting labels from these systems. Figures S1 and S2 show the results of this case study. While we did not create a GUI system yet,

we colored the results manually to match the color system of PubTator. Specifically, we used orange, green, and purple highlights to annotate the predicted disease, chemical, and gene entities, respectively. Additionally, we used blue highlight to point out the ambiguous entities in our system.

Figure S1 shows that BioBBC correctly detected more biomedical entities than PubTator. Specifically, for disease entities, PubTator annotated the names of the diseases “Type 2 Diabetes Mellitus” and “Alzheimer's disease”, but it ignored the mention of their abbreviations “T2D” and “AD”. In contrast, BioBBC detected all the mentions of the diseases and their abbreviation. Additionally, for chemical entities, PubTator wrongly annotated “DEGs” and “hsa-mir-129-2-3p” as chemical components, as the “DEGs” is a general term and “hsa-mir-129-2-3p” should be annotated as a gene. Finally, for the gene entities, as seen, while PubTator annotated some microRNAs as genes, it missed many other entities. In constraints, BioBBC successfully detected all the gene entities with correct boundaries. However, here we notice that while BioBBC has correctly detected the entity “hsa-mir-103a-3p” as a gene, it had wrongly detected the token “103a”, highlighted in blue, as a chemical entity when we tested this text in our chemical model.

Example 2 (Figure S2) shows that BioBBC and PubTator recognized the disease entities correctly. For genes, while PubTator detected FGFR as a gene, it missed many mentions of this gene. BioBBC correctly detected all the gene entities. Moreover, BioBBC detected the entity “Fibroblast growth factor receptors” and “tyrosine kinase” as gene entities. However, in this example, BioBBC also annotated “tyrosine” as a chemical entity. For the chemicals, PubTator detected the first occurrence of the chemical entity “PD173074” and missed the second one. This example demonstrates a PubTator’s consistency problem that is not apparent for BioBBC, which correctly detected all the occurrences of the chemical entities.

The examples in the case study section show how our system BioBBC improves the performance of BioNER by correctly detecting and annotating biomedical entities. Moreover, compared with PubTator, our model, BioBBC, performs better in recognizing more biomedical entities and understanding the structure of the text by effectively learning syntactic and semantic features. Lastly, our model efficiently recognizes the entity boundaries, abbreviations of biomedical entities and avoids inconsistency problems. These advantages demonstrate the benefit of BioBBC in solving BioNER tasks.

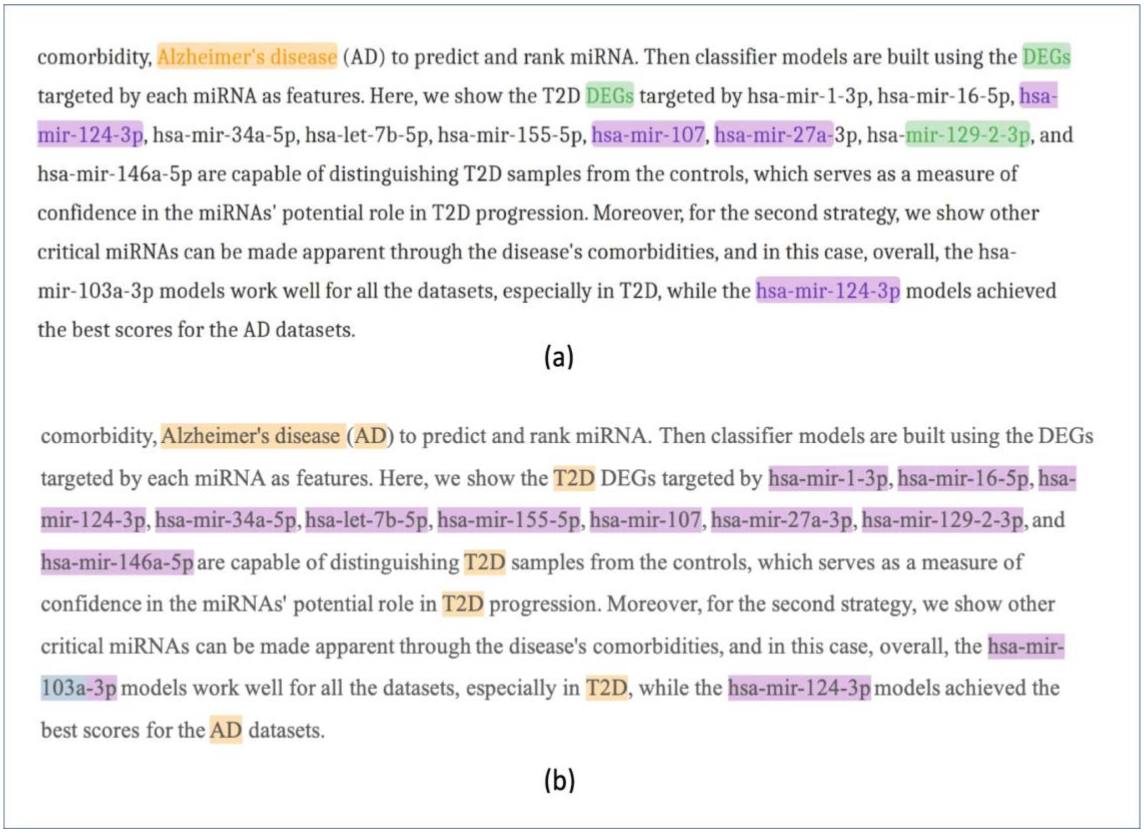

comorbidity, Alzheimer's disease (AD) to predict and rank miRNA. Then classifier models are built using the DEGs targeted by each miRNA as features. Here, we show the T2D DEGs targeted by hsa-mir-1-3p, hsa-mir-16-5p, hsa-mir-124-3p, hsa-mir-34a-5p, hsa-let-7b-5p, hsa-mir-155-5p, hsa-mir-107, hsa-mir-27a-3p, hsa-mir-129-2-3p, and hsa-mir-146a-5p are capable of distinguishing T2D samples from the controls, which serves as a measure of confidence in the miRNAs' potential role in T2D progression. Moreover, for the second strategy, we show other critical miRNAs can be made apparent through the disease's comorbidities, and in this case, overall, the hsa-mir-103a-3p models work well for all the datasets, especially in T2D, while the hsa-mir-124-3p models achieved the best scores for the AD datasets.

(a)

comorbidity, Alzheimer's disease (AD) to predict and rank miRNA. Then classifier models are built using the DEGs targeted by each miRNA as features. Here, we show the T2D DEGs targeted by hsa-mir-1-3p, hsa-mir-16-5p, hsa-mir-124-3p, hsa-mir-34a-5p, hsa-let-7b-5p, hsa-mir-155-5p, hsa-mir-107, hsa-mir-27a-3p, hsa-mir-129-2-3p, and hsa-mir-146a-5p are capable of distinguishing T2D samples from the controls, which serves as a measure of confidence in the miRNAs' potential role in T2D progression. Moreover, for the second strategy, we show other critical miRNAs can be made apparent through the disease's comorbidities, and in this case, overall, the hsa-mir-103a-3p models work well for all the datasets, especially in T2D, while the hsa-mir-124-3p models achieved the best scores for the AD datasets.

(b)

Figure S1. Example 1 of paragraph annotation using a) PubTator, and b) BioBBC

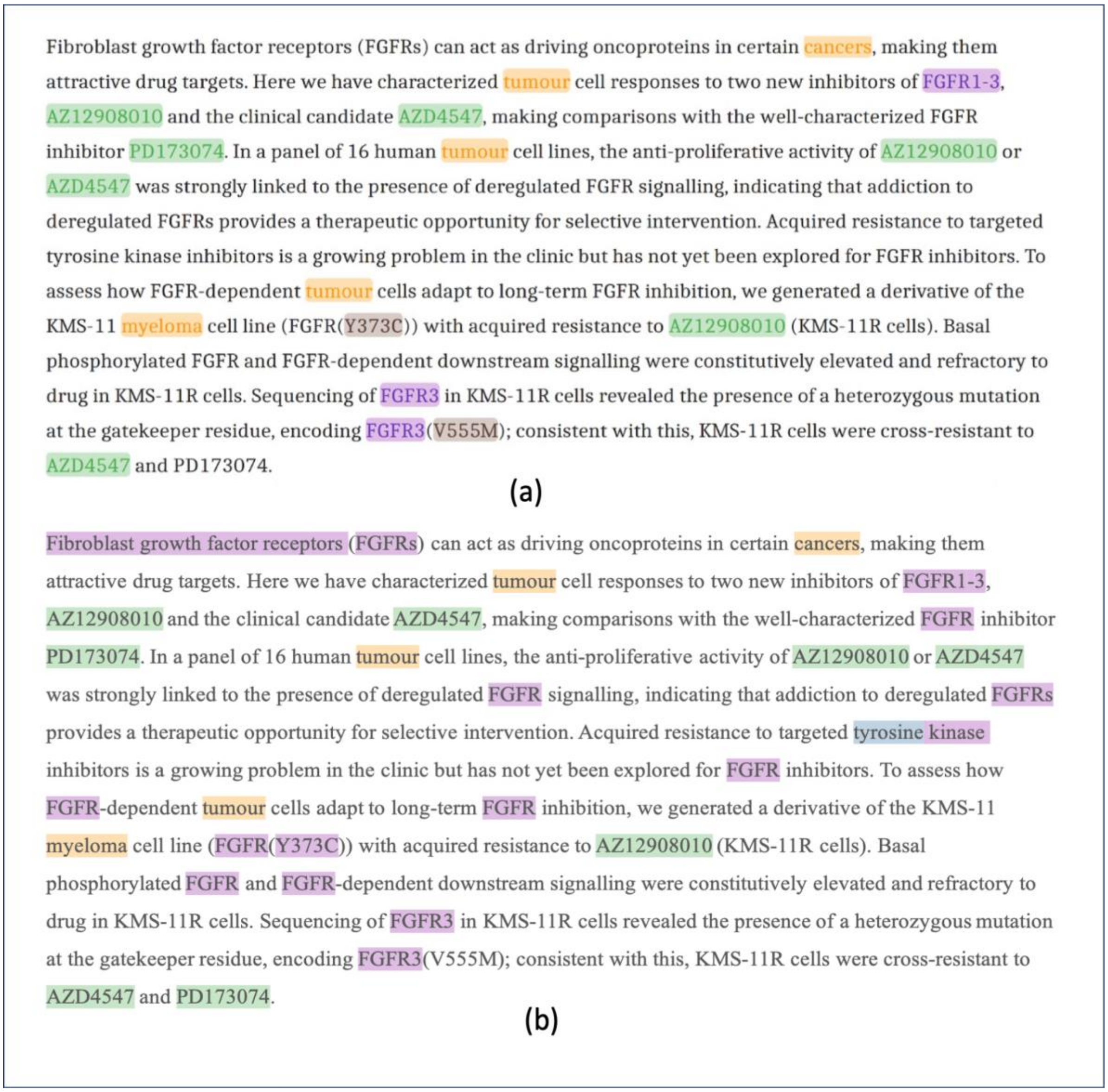

Fibroblast growth factor receptors (FGFRs) can act as driving oncoproteins in certain cancers, making them attractive drug targets. Here we have characterized tumour cell responses to two new inhibitors of FGFR1-3, AZ12908010 and the clinical candidate AZD4547, making comparisons with the well-characterized FGFR inhibitor PD173074. In a panel of 16 human tumour cell lines, the anti-proliferative activity of AZ12908010 or AZD4547 was strongly linked to the presence of deregulated FGFR signalling, indicating that addiction to deregulated FGFRs provides a therapeutic opportunity for selective intervention. Acquired resistance to targeted tyrosine kinase inhibitors is a growing problem in the clinic but has not yet been explored for FGFR inhibitors. To assess how FGFR-dependent tumour cells adapt to long-term FGFR inhibition, we generated a derivative of the KMS-11 myeloma cell line (FGFR(Y373C)) with acquired resistance to AZ12908010 (KMS-11R cells). Basal phosphorylated FGFR and FGFR-dependent downstream signalling were constitutively elevated and refractory to drug in KMS-11R cells. Sequencing of FGFR3 in KMS-11R cells revealed the presence of a heterozygous mutation at the gatekeeper residue, encoding FGFR3(V555M); consistent with this, KMS-11R cells were cross-resistant to AZD4547 and PD173074.

(a)

Fibroblast growth factor receptors (FGFRs) can act as driving oncoproteins in certain cancers, making them attractive drug targets. Here we have characterized tumour cell responses to two new inhibitors of FGFR1-3, AZ12908010 and the clinical candidate AZD4547, making comparisons with the well-characterized FGFR inhibitor PD173074. In a panel of 16 human tumour cell lines, the anti-proliferative activity of AZ12908010 or AZD4547 was strongly linked to the presence of deregulated FGFR signalling, indicating that addiction to deregulated FGFRs provides a therapeutic opportunity for selective intervention. Acquired resistance to targeted tyrosine kinase inhibitors is a growing problem in the clinic but has not yet been explored for FGFR inhibitors. To assess how FGFR-dependent tumour cells adapt to long-term FGFR inhibition, we generated a derivative of the KMS-11 myeloma cell line (FGFR(Y373C)) with acquired resistance to AZ12908010 (KMS-11R cells). Basal phosphorylated FGFR and FGFR-dependent downstream signalling were constitutively elevated and refractory to drug in KMS-11R cells. Sequencing of FGFR3 in KMS-11R cells revealed the presence of a heterozygous mutation at the gatekeeper residue, encoding FGFR3(V555M); consistent with this, KMS-11R cells were cross-resistant to AZD4547 and PD173074.

(b)

Figure S2. Example 2 of paragraph annotation using a) PubTator, and b) BioBBC.

- ***Example of error cases produced by BioBBC***

Here, we present some error cases produced by BioBBC (see Table S2). In the first sentence, the model wrongly annotated the term ‘cocaine’ as a disease. In the second example, while the term ‘amino acid’ is detected as chemical, the model missed its abbreviation ‘AA’. In the third example, the word ‘reduced’ is captured as the first word of the entity; the boundary expansion in this example may occur due to the syntactic information captured in our model, which denotes the

word reduced as a part of the phrase. In the last example, the term ‘insulins’ was wrongly detected as a gene.

Table S2. Example of error cases.

Text	Entity	Error explanation
metabolite alterations in stimulant (methamphetamine and **cocaine**) substance use disorders (suds) for over 25 years	cocaine	Detected as Disease - Wrong annotation.
comparisons between their amino acid (AA) sequences and other known	(AA)	Chemical - Missed annotation
**reduced nicotinamide adenine dinucleotide phosphate - diaphorase** (**nadph - d**) histochemistry was also employed to	reduced	Detected as Chemical - Wrong annotation
of a combination with once - weekly **insulins** that have	insulins	Detected as Gene - Wrong annotation

The error cases show room for improvement in the BioNER. Specifically, the information captured in our model could be improved since the syntactic information of the complex biomedical sentences was extracted using a general domain tool. Also, using one-hot encoding for the POS tags may have some limitations. However, we conducted a preliminary experiment to compare one-hot vectors with Glove embeddings and observed a decrease in performance when using Glove. This outcome suggests that further configuration might be necessary. The drop in performance could be attributed to our initial optimization for one-hot encoders, indicating that our parameters and settings may need further adjustment to use other embedding methods.

References

1 Alamro, H. *et al.* Type 2 Diabetes Mellitus and its comorbidity, Alzheimer's disease: Identifying critical microRNA using machine learning. *Front. Endocrinol.* **13**, 1084656 (2022). https://doi.org:10.3389/fendo.2022.1084656

2 Chell, V. *et al.* Tumour cell responses to new fibroblast growth factor receptor tyrosine kinase inhibitors and identification of a gatekeeper mutation in FGFR3 as a mechanism of acquired resistance. *Oncogene* **32**, 3059-3070 (2013). https://doi.org:10.1038/onc.2012.319
